# Adverse Audio-Vestibular Effects of Drugs and Vaccines Used in the Treatment and Prevention of COVID-19: A Review

**DOI:** 10.3390/audiolres12030025

**Published:** 2022-04-29

**Authors:** Magdalena B. Skarzynska, Monika Matusiak, Piotr H. Skarzynski

**Affiliations:** 1 Institute of Sensory Organs, 05-830 Warsaw, Poland; p.skarzynski@csim.pl; 2 Center of Hearing and Speech Medincus, 05-830 Warsaw, Poland; 3 World Hearing Center, Oto-Rhino-Laryngology Surgery Department, Institute of Physiology and Pathology of Hearing, 05-830 Warsaw, Poland; m.matusiak@ifps.org.pl; 4 World Hearing Center, Department of Teleaudiology of Hearing, Institute of Physiology and Pathology of Hearing, 05-830 Warsaw, Poland; 5 Heart Failure and Cardiac Rehabilitation Department, Faculty of Medicine, Medical University of Warsaw, 03-242 Warsaw, Poland

**Keywords:** ototoxicity, COVID-19, vaccine, hearing loss, tinnitus, dizziness, audio-vestibular side-effects

## Abstract

(1) Background: The purpose of this article is to review pharmacological treatments for COVID-19 (currently approved by the EMA (European Medical Agency) and FDA (Food and Drug Administration)) and highlight their potential audio-vestibular side-effects as an ototoxic adverse reaction. (2) Methods: Review of the available literature in the scientific databases PubMed, ResearchGate, Scopus, and ScienceDirect, and in summaries of product data sheets. (3) Results: In accordance with EBM (evidence-based medicine) the treatment of COVID-19 by using lopinavir/ritonavir, chloroquine and hydroxychloroquine, azithromycin, favipiravir, amantadine, oseltamivir, and ivermectin is no longer recommended for patients suffering from COVID-19 due to a lack of clinical data, publications, and recommendations. There were 39 publications and 15 summaries of product characteristics (as other sources of data) which were also used in this analysis. Adverse events could be permanent or disappear over time. Following treatment for COVID-19, the most frequent adverse audio-vestibular reactions reported in clinical trials and publications in the area of audiology and otorhinolaryngology were: dizziness, blurry vision with dizziness, nasopharyngitis, dysgeusia, and tinnitus. As far as vaccines are concerned, dizziness as an ototoxic effect was uncommon and occurs only in hypersensitive people who experience anaphylactic shock. (4) Conclusions: The ototoxicity of the drugs discussed here does not have as severe symptoms as the drugs used in the treatment of COVID-19 in 2020 (e.g., hydroxychloroquine), and relates mainly to disorders of the vestibulocochlear system. However, there is still a need to monitor ototoxic side-effects because of potential interactions with other ototoxic drugs. Many of the drugs approved by EMA and FDA are new, and not every side-effect is known.

## 1. Introduction

Reports on the pharmacotherapy of COVID-19 patients have been presented over the past year, and they indicate two main processes involved in the pathogenesis of the disease. In the early stages of infection, the disease is driven primarily by the replication of severe acute respiratory syndrome coronavirus 2 (SARS-CoV-2). Later in the course of infection, the disease is fueled by an excessive immune/inflammatory response of the virus, leading to tissue damage. Based on this understanding, it is expected that antiviral therapies will have the greatest impact on the early stages of the disease, while immunosuppressive/anti-inflammatory therapies are likely to be more beneficial in the later stages so as to prevent an outbreak of a “cytokine storm”. Due to the experimental treatments used in clinical trials, the treatments may carry the risk of side-effects, in particular audio-vestibular effects [1]. The purpose of this review is to update information on the new coronavirus that is important from a therapeutic perspective, in particular the risk of ototoxicity and of inducing audiological or vestibular disorders arising from adverse reactions to drugs currently used in COVID-19 pharmacotherapy.

There are now several treatment models for COVID-19 approved by the FDA (Food and Drugs Administration) for use in the U.S. and by the EMA (European Medical Agency) for use in the E.U. In early December 2020, the FDA approved a vaccine to prevent disease development [2]. In the early stages of infection, the disease develops through replication of SARS-CoV-2 virus cells, before the host organism can produce an effective immune response, so at this time the best therapy model is probably an anti-SARS-CoV-2 antibody-based therapy. On 9 November 2020, the FDA granted Emergency Approval (EUA) authorizing of the administration of bamlanivimab for the treatment of mild to moderate coronavirus (COVID-19) in adults and pediatric patients with positive direct SARS-CoV-2 viral tests in those 12 years of age or older, weight of at least 40 kg, and at high risk of progression to severe COVID-19 and/or hospitalization [2,3,4]. On November 19, a decision was issued allowing the use of baricitinib in combination with remdesivir in the treatment of suspected or laboratory-confirmed 2019 coronavirus disease (COVID-19) in hospitalized patients 2 years of age and older who required supplemental oxygen, invasive mechanical ventilation, or extracorporeal membrane oxygenation (ECMO) [2,5]. Two days later, on 21 November 2020, the combination of casirivimab and imdewimab was approved for emergency use.

The anti-SARS-CoV-2 monoclonal antibodies, bamlanivimab and casirivimab, in combination with imdevimab, are available under emergency authorization for outpatients at high risk of disease progression [6,7,8]. Remdesivir is recommended for hospitalized patients who require supplemental oxygen. However, administration of this drug is not routinely recommended in patients who require mechanical ventilation, as there is limited evidence of a benefit of this drug in advanced disease [9,10,11,12]. Additionally, it was found that dexamethasone—a corticosteroid drug—improves the survival of hospitalized patients who require supplemental oxygen. Patients requiring mechanical ventilation show the greatest benefits [13]. In 2021, the FDA and the EMA authorized a few new drugs for “in emergency use” or “additional monitored” in treating COVID-19: anakinra, regdanvimab, tocilizumab, casirivimab/imdevimab, sotrovimab, and baricitinib. Tixagevimab/ cilgavimab is currently under rolling review by the EMA. Due to the greater number of options for treating COVID-19 approved by the EMA and FDA in 2020 and 2021, only these drugs are discussed here.

Ototoxic drugs can damage the inner ear through several common mechanisms, resulting in a sudden increase in hearing threshold or a loss of hearing. Ototoxicity is defined as an adverse pharmacological temporary or long-lasting reaction that can affect the auditory nerve or the inner ear. It is characterized by cochlear or vestibular dysfunction and can have long-lasting consequences for the future quality of life of the patient, although in some cases the benefits and medical necessity for using a drug outweigh the risk of loss of hearing [14]. Ototoxicity is a side-effect that is a risk for the entire population, but some sections of society are particularly vulnerable, such as the elderly and young children.

According to clinical protocols and the recommendations of scientific societies, monitoring for early detection of ototoxicity allows treatment regimens to be changed and hence can minimize, or even prevent, ototoxicity and its accompanying balance impairment or hearing loss [14,15,16]. According to the American Academy of Audiology (AAA), sensorineural degradation and auditory damage can lead to permanent hearing loss and tinnitus. Audiological monitoring for ototoxicity should be performed for a number of reasons. First, audiological testing allows hearing impairment to be detected as soon as possible before a severe handicap occurs. Second, early detection of a change in hearing may lead to reconsideration of the drug regimen [17]. Third, it is important to bear in mind that a drug may become ototoxic not alone but in combination with a number of other drugs, so that a cumulative adverse effect may build up. The final side-effect of ototoxicity depends on the: (1) the route of administration, (2) duration of the therapy, (3) sensitivity of the patient to the ototoxic drug, (4) dosage and infusion rate, (5) renal and hepatic function, and (6) genetic predisposition. Additionally, the longer the therapy goes on, the higher the risk of ototoxicity.

The audio-vestibular side-effects of drugs are classified as ototoxic side-effects. Tinnitus, vertigo, and dizziness are examples of audio-vestibular side-effects that should be considered during treatment for COVID-19. Otorhinolaryngologists, neurologists, audiological specialists, and general practitioners should be aware of this type of side-effect. Tinnitus may manifest as sensorineural hearing loss (SNHL) or may be a consequence of central alterations caused by drugs. Vestibular side-effects may manifest as loss of balance and dizziness, instability, unsteadiness, and difficulties in maintaining an upright posture [18,19]. Audio-vestibular disorders should be considered when, during treatment, COVID-19 patients require more than one treatment option or are being treated for additional comorbidities [19]. According to the literature and clinical observations, COVID-19 may cause hearing loss, and this may be an important factor when diagnosing audio-vestibular disorders. Consequently, according to the known clinical effects in human and animal models, health care providers should monitor signs and symptoms of audio-vestibular disorders or impairment in COVID-19 patients and are being treated with drugs against the virus [20,21,22,23,24,25,26,27].

## 2. Materials and Methods

**Figure 1:**
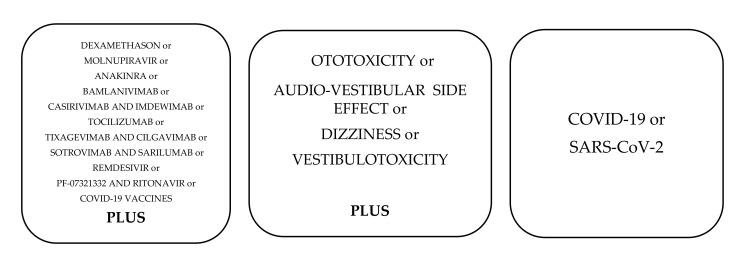
The combinations of words used for searching articles.

**Figure 2:**
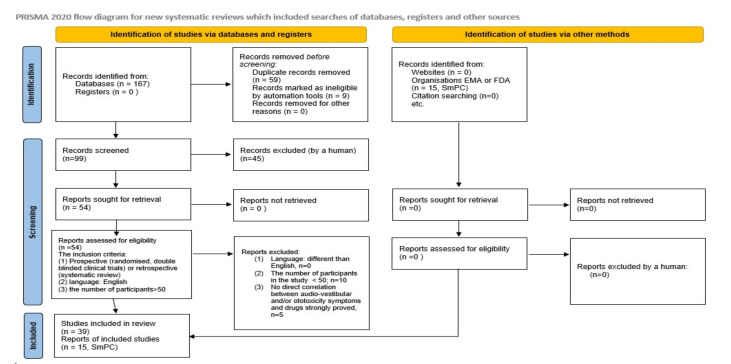
PRISMA flow diagram, outlining the identification, screening, eligibility, and inclusion criteria of the review [28].

A database search was conducted to identify the relevant literature. This involved Medline, US National Library of National Institutes of Health (PubMed), ResearchGate, Scopus, and ScienceDirect, as well as the clinical trials database and google scholar. There was no limit to the time-frame of the search. The search was conducted using the terms: ototoxicity, vestibulotoxicity, audio-vestibular side-effect, dizziness, COVID-19, SARS-CoV-2, dexamethasone; molnupiravir, anakinra, bamlanivimab, casirivimab and imdewimab, tocilizumab, tixagevimab and cilgavimab, sotrovimab and sarilumab, PF-07321332 and ritonavir, and COVID-19 vaccines. Combinations of words that were searched are shown in Figure 1. The records were reviewed by title and abstract, analyzing the full texts if there were any doubts as to the suitability of the work for inclusion. After excluding articles relating to treatments not covered by this article and which were outside its scope, 39 articles were considered appropriate for this scoping review as well as 15 summaries of product characteristics in accordance with the PRISMA statements (Figure 2). The inclusion criteria for searching were: (1) prospective (randomized, double-blinded clinical trials) or retrospective (systematic review); (2) language: English; (3) the number of participants in the study > 50 (there were no age criteria in the inclusion criteria, noting that some medicines (e.g., casirivimab and imdevimab) are approved not only for adults but also for children over 12 years old; similarly, vaccines have the same status—approved for prevention of COVID-19 in children over 5 years old). The exclusion criteria were: (1) language: other than English; (2) the number of participants in the study < 50; (3) no direct correlation between audio-vestibular and/or ototoxicity symptoms and the approved drug.

## 3. Results

The summary of potential ototoxicity and audio-vestibular disorders, which was based on 39 publications and 15 summaries of product characteristics, were included into this analysis are classified in the Table 1 and discussed below. The drugs that have been identified during literature search: anakinra, tocilizumab, COVID-19 vaccines, remdesivir, tocilizumab, dexamethasone, molnupiravir, ritonavir, tixagevimab, and cilgavimab. The drugs that have been identified during searching of the summaries of the product characteristics: dexamethasone, molnupiravir, anakinra, bamlanivimab, casirivimab and imdewimab, tocilizumab tixagevimab and cilgavimab, sotrovimab and sarilumab, remdesivir, pf-07321332 and ritonavir and COVID-19 vaccines. The possible overlaps from publications and summaries of product characteristics are based mainly on the mechanism of actions of each of the drugs and the side-effects of the COVID-19 vaccines and drugs. All drugs were divided into three categories: (1) monoclonal antibodies and other anti-COVID-19 drugs, (2) COVID-19 vaccines, and (3) anti-COVID-19 oral treatment. Each drug and vaccine were described by the mechanism of action, clinical trials, and possible side-effects with emphasis on the audio-vestibular side-effects and ototoxicity. The classification is presented in Table 1. While the information about the side-effects was limited or when there was no information about the ototoxicity and audio-vestibular disorders, we also wrote it in the Table 1. Additionally, the searching of audio-vestibular disorders caused by COVID-19 was searching by using the same combination the words: ototoxicity or audio-vestibular side-effects or dizziness or vestibulotoxicity and COVID-19 or SARS-CoV-2. There were differences between the adverse reactions in the area of ENT reported in publications and summaries of product characteristics. An example was COVID vaccines, where dizziness was reported as a side-effect, but in the summary of product characteristics, the dizziness was described as a part of anaphylactic shock.

### 3.1. Anti-COVID-19 Drugs

#### 3.1.1. Dexamethasone

Dexamethasone (DEX) is a steroid compound that belongs to the group of corticosteroids (more precisely, glucocorticoids). This treatment group is used to treat many diseases and symptoms including chronic obstructive pulmonary disease, severe allergies, rheumatic problems, asthma, several skin conditions, cerebral edema, and, together with antibiotics, for tuberculosis [29,30]. Dexamethasone has an anti-inflammatory effect and is therefore mainly used as an adjunct in the treatment of viral pneumonia. Dexamethasone is similar to compounds endogenously produced by the body to inhibit inflammation. However, this effect is approximately 25 times more potent than other corticosteroid compounds. Hence, due to its higher potency, it is suspected that this drug might prove effective in treating patients with SARS-CoV-2 [30,31,32]. In addition, dexamethasone is also more potent than non-steroidal anti-inflammatory drugs (NSAIDs) such as ibuprofen and aspirin and has both anti-inflammatory and immunosuppressive effects, while NSAIDs only inhibit the vascular phase of inflammation. The mechanism of action of DEX is mediated by inhibition of the pro-inflammatory gene that codes for chemokines, cytokines, cell adhesion molecules (CAM), and acute inflammatory response. The anti-inflammatory effect takes place in two ways: chemotaxis and vasodilation [30,33,34]. Coronaviruses activate aryl hydrocarbon receptors (AhR) after entering their target cells through a mechanism independent of indoleamine 2,3-dioxygenase (IDO1). The activated AhR induces the upregulation of a series of AhR-dependent effectors downstream, resulting in the “Systemic AhR Activation Syndrome” (SAAS). SAAS includes inflammation, thromboembolism, and fibrosis, with the consequent damage to many organs and their potential death. The use of dexamethasone is possible because, as shown in studies, dexamethasone appears to downgrade both AhR and IDO1 genes, thus further reducing inflammation [30,35,36]. The use of dexamethasone in this setting is strongly recommended as patients with severe COVID-19 may develop a systemic inflammatory response that may lead to lung damage and multi-organ dysfunction [37]. The EMA endorsed the use of dexamethasone in COVID-19 patients undergoing aerobic or mechanical ventilation [38]. As recommended by the EMA, dexamethasone has been included in treatment protocols for patients diagnosed with COVID-19. However, side-effects such as increased appetite, mood changes, agitation, and headache are possible as a result of treatment with the drug. It sometimes causes blurry vision with dizziness, and in the long-term (over a week) can lead to arrhythmias [30,37].

#### 3.1.2. Anakinra

Anakinra has been authorized for treatment of many diseases such as rheumatoid arthritis for adult patients, autoinflammatory periodic fever syndromes, and for treatment of Cryopyrin-Associated Periodic Syndromes. In 2021 anakinra was approved by EMA for treatment of coronavirus disease—COVID-19, for adult patients who suffer from pneumonia and requiring supplemental oxygen and those who are at risk of progressing to a severe state of respiratory failure. The mechanism of action of anakinra is based on neutralization of the biologic activity of interleukins (IL-1α and IL-1β) by inhibiting the binding to the receptor IL-1RI. In total, 405 patients were enrolled to the randomized placebo-controlled clinical trial (SAVE-MORE study) and the efficacy of anakinra was investigated. No different side-effects were found during this clinical study. Many severe side-effects may be observed following administration of anakinra (e.g., serious infections, neutropenia, thrombocytopenia, non-infectious hepatitis), but none of the side-effects may be classified as an audio-vestibular disorder [39,40].

### 3.2. Monoclonal Antibodies

The need to develop new effective therapies has contributed to an intensive search for pharmacotherapy methods for treatment of COVID-19 disease, including research on existing drugs with specific indications, including the parallel development of new therapies. The possibility of using monoclonal antibodies has been pointed out. Historically, monoclonal antibody therapies have found many applications. They have been used in viral infections such as pneumonia with respiratory syncytial virus, as well as in the case of the Ebola virus. Early models of therapy were designed to contain “the cytokine storm”, as observed with varying levels of efficacy for the drugs under study. Next, attention was drawn to the possibility of using plasma from convalescents, for whom it is assumed that the active substances are neutralizing polyclonal antibodies [41,42]. Since the onset of the SARS-CoV-2 pandemic, biopharmaceutical companies and academic scientists have collaborated to extract neutralizing monoclonal antibodies from convalescents. It involves a multi-step process, including selection of peripheral blood mononuclear cells from plasma donors, isolation of receptor-specific binding domains, single memory B cells, cloning, transfection, and finally production of a monoclonal antibody. Thanks to this research methodology, many monoclonal antibodies with potential neutralizing effects against SARS-CoV-2 have been discovered, but only a few are currently being tested in clinical trials or have been approved for COVID-19 treatment [43].

#### 3.2.1. Bamlanivimab

Bamlanivimab is a recombinant, fully neutralizing human IgG 1 mAb antibody. Specifically, this mAb was selected as the one with the best binding affinity for SARS-CoV-2 out of over 500 antibodies; it targets the RBD of the spearhead protein. On 10 November 2020, the U.S. Food and Drug Administration (FDA) granted emergency approval for bamlanivimab for the treatment of mild to moderate COVID-19 in adult and paediatric patients. This is the first nAb approved for clinical use [8]. The ototoxicity of bamlanuvimab has not been clearly indicated, and data indicating a possible ototoxic effect of the drug remain limited. However, reports from clinical studies, including studies in COVID-19 patients, suggest that dizziness may occur in patients treated with bamlanivimab. In a BLAZE-1 clinical trial, 3.2% of patients reported an adverse event of dizziness [3,44,45,46].

#### 3.2.2. Casirivimab and Imdewimab

Casirivimab and imdevimab are experimental therapies and have been approved by the FDA for use in emergencies. Imdewimab and casirivimab are human immunoglobulin G1 (IgG1) antibodies that reduce endogenous immunoglobulin G levels by up to 79% [47,48]. Casirivimab and imdevimab is a combination therapy consisting of two monoclonal antibodies made by Regeneron. The emergency approval of this mixture was approved based on the results of a clinical trial of 799 non-litigated adults with mild to moderate symptoms of COVID-19. Participants were divided into three groups, two of which received a combination of casirivimab and imdevimab, but in different doses; the third group received a placebo. For patients at high risk of developing severe disease, those treated with monoclonal antibodies had a reduced risk (3% versus 9%) of hospitalization or an emergency room visit within 28 days of starting treatment [7,49,50,51].

#### 3.2.3. Tocilizumab and Sarilumab

The efficacy of these interleukin-6 receptor antagonists, including improved outcome and survival, in severe and critically ill patients with COVID-19 who were receiving organ support in intensive care with treatment with the tocilizumab and sarilumab (IL-6 receptor antagonists), was assessed in 803 adult patients: 353 patients received tocilizumab, 48 sarilumab, and 402 were enrolled to the control group. According to the results of this preliminary study, the combination of these drugs reduced the death rate from nearly 36% in the control group to 28% among those patients who received tocilizumab and to 22% for those who received sarilumab [52,53]. Tocilizumab is an immunosuppressive drug which binds specifically to both soluble and membrane-bound IL-6 receptors (sIL-6R and mIL-6R) and inhibits sIL-6R and mIL-6R-mediated signaling. IL-6 is a pleiotropic pro-inflammatory cytokine produced by a variety of cell types including T- and B-cells, monocytes, and fibroblasts and is involved in diverse physiological processes such as T-cell activation, induction of immunoglobulin secretion, induction of hepatic acute phase protein synthesis, and stimulation of hematopoiesis. Dizziness, as a type of ototoxicity side-effect, is classified as common [54,55]. Sarilumab is a human monoclonal antibody (IgG1 subtype) that specifically binds to both soluble and membrane-bound IL-6 receptors (IL-6Rα) and inhibits IL-6-mediated signaling which involves ubiquitous signal-transducing glycoprotein 130 (gp130) and the Signal Transducer and Activator of Transcription-3 (STAT-3). The activity of sarilumab reduces inflammation associated with laboratory changes such as a decrease in ANC and an elevation in lipids. According to the clinical data obtained from clinical trials, in the ENT (ear-nose-throat) area, only nasopharyngitis was reported as a common side-effect. Ototoxicity was not reported as a side-effect [52,56,57].

#### 3.2.4. Tixagevimab/Cilgavimab

Tixagevimab and cilgavimab are both recombinant human IgG1κ monoclonal antibodies and the mechanism of action against SARS-CoV-2 is based on them binding to non-overlapping regions of receptor binding domain (RBD) of spike proteins. In total, 4220 subjects were enrolled in two clinical studies (PROVENT and STORM CHASER), where safety and efficiency were examined. In the area of audio-vestibular disorders, no side-effects have so far been identified [58,59].

#### 3.2.5. Sotrovimab

Sotrovimab is a monoclonal antibody (IgG1κ) and is indicated for the treatment of adults and adolescents above 12 years and weighing more than 40 kg, suffering from COVID-19 disease, not requiring supplemental oxygen but at the same time are at risk of progressing to severe phase COVID-19. The adverse reactions following administration of sotrovimab are rare or uncommon and include hypersensitivity reactions (rash, bronchospasm), infusion-related reactions, and anaphylaxis, and dyspnea, but none of these can be classified as audio-vestibular disorders. The clinical efficacy was studied in a phase II/III randomized, double-blind, and placebo-controlled clinical trial for treatment of COVID-19 in 1057 non-hospitalized, non-vaccinated adult patients (COMET-ICE clinical trial). The adjusted relative risk reduction in hospitalization or death at 29 days of observation was 79% [60,61].

### 3.3. Oral Treatments against COVID-19

#### 3.3.1. Molnupiravir

Molnupiravir is a prodrug. It is a nucleotide analogue that inhibits SARS-CoV-2 replication by viral mutagenesis and is the 5’-isobutyrate ester of the ribonucleotide analogue N4-hydroxycytidinean, an orally administered inhibitor of replication of the SARS-CoV-2 virus [62]. The indication for treatment is mild-to-moderate COVID-19 in patients < 18 years old who have a positive result from viral testing of direct severe acute respiratory syndrome coronavirus 2 (SARS-CoV-2), as well as those who are at high risk for progressing to severe COVID-19. This drug was approved by the FDA in 2021. The safety of molnupiravir at a dose of 800 mg twice a day for 5 days has been assessed in a clinical trial (phase 3, double-blind, acronym MOVe-OUT) where 1411 of non-hospitalized subjects with COVID-19 were randomly divided into two groups: *N* = 710 subjects treated with molnupiravir and *N* = 701 subjects treated with placebo. According to the results of the study, serious adverse effects occurred in 7% of the subjects who received molnupiravir and in 10% of those who received placebo. The most common adverse reaction reported in the area of audiology side-effects was dizziness, which occurred in 1% of patients who received molnupiravir or placebo. Other side-effects were diarrhea and nausea [62,63].

#### 3.3.2. PF-07321332/Ritonavir (Brand Name: Paxlovid)

Paxlovid is the brand name of co-packaged tablets PF-07321332/ritonavir. PF-07321332 is a SARS-CoV-2 main protease (Mpro) inhibitor, and ritonavir is an HIV-1 protease inhibitor and CYP3A inhibitor [64]. The dosage scheme of oral co-administration is 300 mg (two tablets of 150 mg each) of PF-07321332 and 100 mg (one tablet) of ritonavir twice a day for 5 days. The safety and adverse reactions have been assessed in a clinical trial (phase 2/3, randomized, placebo-controlled (C4671005 EPIC-HR)). 2224 adult subjects were enrolled and divided into two groups: Paxlovid group (*N* = 1109) and placebo (*N* = 1115). The most common adverse reactions were diarrhea, hypertension, and myalgia. One otorhinolaryngology-related side-effect reported after administration of Paxlovid was dysgeusia. It was reported in 6% of subjects from the Paxlovid group and in 1% of the placebo group [64].

### 3.4. COVID-19 Vaccines

At the same time as developing new therapeutic approaches to treating COVID-19, researchers around the world have focused on vaccine development [65,66]. As of January 2021, two vaccines, the Pfizer-BioNTech vaccine and the Moderna vaccine, have been approved for marketing. In addition, AstraZeneca was approved for use in the UK at the end of December 2020—at first for emergency use and then for regular use. At the end of 2021, the vaccine from the Novavax company was also approved for use, and work on further preparations is in progress [67,68]. Clinical trials show that systemic and local reactions may occur as a result of vaccination. Patients most frequently report pain at the injection site, and less frequently redness or swelling at the site. Systemically, patients most often report headache and fatigue, and less frequently fever. In the Pfizer-BioNTech study, four serious adverse events were considered vaccine-related, and two non-vaccine-related deaths were reported in the vaccine group. In the Moderna study, six cases of adverse events were related to the study intervention, and two fatalities were reported in the study group, which were considered unrelated to the drug. The incidence of serious adverse events in the Moderna study was similar in the study and control groups. Both study sponsors will continue to follow the safety and efficacy of the vaccine for the next 2 years [69,70].

After COVID-19 vaccines are approved and marketed, safety authorities continue to monitor and analyze possible adverse events. This process ensures that the benefits continue to outweigh the risks for those receiving the vaccines. Such continuous monitoring for side-effects can detect adverse events that may not have occurred in clinical trials but which may occur when the vaccine is administered to a larger population. Information is needed on possible long-term side-effects, including potential ototoxicity, as there has been a rapid development of vaccines and conditional approval without the long-term effects being investigated [67,68,71]. Some authors consider that there is a cause–effect relationship between Sudden Sensorineural Hearing Loss (SSNHL) or tinnitus and vaccines, but this relationship has been described as indirect, and new studies and observations need to be carried out [23,26,72].

#### 3.4.1. Pfizer + BioNTechVaccine: Comirnaty

According to the summary of product characteristics, Comirnaty comes as a concentrate for injection in a multi-dose vial and must be diluted before use. One vial (0.45 mL) contains six doses of 0.3 mL after dilution, so that one dose contains 30 µg of COVID-19 mRNA embedded in lipid nanoparticles. The safety of Comirnaty was evaluated in participants enrolled in two clinical studies that included 21,744 participants who had received at least one dose of the vaccine. The most frequent reported adverse reactions in participants were: pain at the injection site (>80%), fatigue (>60%), headache (> 50%), myalgia and chills (>30%), arthralgia (>20%), pyrexia, and swelling at the injection site (>10%). The side-effects were usually mild to moderate and usually resolved within a few days after vaccination. Dizziness as an ototoxic effect occurred only in hypersensitive people as part of anaphylactic shock, and its frequency is unknown (it cannot be estimated from available data) [73].

#### 3.4.2. COVID-19 Vaccine: Moderna

According to the summary of its product characteristics, Moderna COVID-19 vaccine is a dispersion for injection in a multi-dose vial containing 10 doses of 0.5 mL. One vial of 0.5 mL contains 100 µg of COVID-19 messenger RNA embedded in SM-102 lipid nanoparticles. The safety profile has been evaluated in a Phase 3 clinical trial (randomized, placebo-controlled, observer-blind) involving 30,351 participants who received at least one dose of the vaccine. The most frequent reported adverse reactions in participants were: pain at the injection site (92%), fatigue (70%), headache (64.7%), myalgia (61.5%), arthralgia (46.4%), chills (45.4%), nausea/vomiting (23%), axillary swelling/tenderness (19.8%), fever (15.5%), swelling at the injection site (14.7%), and redness (10%). The side-effects were usually mild to moderate and usually resolved within a few days after vaccination. Dizziness as an ototoxic effect may occurred only in hypersensitive people as part of anaphylactic shock, and its frequency is unknown (cannot be estimated from the available data) [74].

#### 3.4.3. COVID-19 Vaccine: Astra Zeneca

According to information for UK healthcare professionals, the AstraZeneca COVID-19 vaccine is a solution for injection in a multi-dose container. One dose (0.5 mL) contains COVID-19 vaccine (ChAdOx1-S*recombinant) made up of 5 × 10^10^ viral particles (vp). The safety profile has been evaluated in 4 clinical trials involving 23,745 participants. The most frequently reported adverse reactions were: injection site tenderness (>60%); injection site pain, headache, fatigue (>50%); myalgia, malaise (>40%); pyrexia, chills (>30%), arthralgia, and nausea (>20%). The side-effects were usually mild to moderate and usually resolved within a few days after vaccination. Dizziness as an ototoxic effect occurred only in hypersensitive people as part of anaphylactic shock, the frequency of which is unknown (cannot be estimated from the available data), as with the other two vaccines [71,75,76].

#### 3.4.4. COVID-19 Vaccine: Janssen

Janssen vaccine is an adenovirus vaccine against COVID-19 disease and one dose (0.5 mL) contains adenovirus type 26 encoding the SARS-CoV-2 spike glycoprotein* (Ad26.COV2-S, not less than 8.92 log10 infectious units). According to the summary of product characteristics, the vaccine is only for adult patients and the primary injection is administered as a single dose of 0.5 mL by intramuscular injection only. A booster dose (second dose) of 0.5 mL may be administered intramuscularly at least 2 months after the primary vaccination. The safety of Janssen COVID-19 vaccine has been evaluated in an ongoing Phase 3 study (COV3001) in which a total of 21,895 adults aged 18 years and older received a single-dose vaccination. The most common systemic adverse reactions were headache (38.9%), fatigue (38.2%), myalgia (33.2%), and nausea (14.2%). Pyrexia (body temperature ≥ 38.0 °C) was observed in 9% of participants. The most important side-effects in the audiology and otorhinolaryngology field reported in the clinical trials were dizziness (of uncommon frequency) and tinnitus (rare).

#### 3.4.5. COVID-19 Vaccine: Novovax (Nuvaxovid)

Nuvaxovid is a recombinant, adjuvanted vaccine against COVID-19 and one dose (0.5 mL) contains 5 µg of the SARS-CoV-2 spike protein and is adjuvanted with Matrix-M. The safety profile was evaluated from five clinical trials involving 49,950 adult participants (Nuvaxovid group *N* = 30,058 and placebo group *N* = 19,892). The adverse reactions were mild to moderate and included site tenderness (75%), injection site pain (62%), fatigue (53%), myalgia (51%), headache (50%), malaise (41%), arthralgia (24%), and nausea and vomiting (15%). The duration of adverse reactions was a few days. No adverse reactions important from an otorhinolaryngological or audiological point of view were reported. Dizziness as an ototoxic effect occurred only in hypersensitive people as part of anaphylactic shock and as an adverse reaction with uncommon frequency [77].

## 4. Discussion

Based on various models of pharmacotherapy, clinical trials are designed and conducted in a way that we can understand treatment mechanisms from a therapeutic perspective. To ensure maximum patient safety, the results from new research provide experimental knowledge that can be used in well-designed studies. From guidelines for managing patients diagnosed with COVID-19, a pharmacotherapy model is selected to allow the patient’s health condition to be classified in terms of their disease symptoms. The drugs used are selected depending on the scale of the disease symptoms [38,94]. Based on EBM (Evidence Based Medicine), the treatment of COVID-19 with lopinavir/ritonavir, chloroquine and hydroxychloroquine, azithromycin, favipiravir, amantadine, oseltamivir, and ivermectin is no longer recommended for treatment of COVID-19 due to a lack of clinical data, publications, and recommendation. Recommendations for treatment depend on the stage of the disease, but, as a standard, only one anti-COVID-19 drug is used at a time. Consequently, this reduces the risk of seeing audio-vestibular disorders, but even still the cause-and-effect relationship between audio-vestibular disorders and taking medication for treatment of COVID-19 or vaccines for COVID-19 needs to be considered. Primary care physicians, as well as ENT specialists, should be aware of the possible connection between these sorts of side-effects and drugs [19]. Many of these drugs are new, and as a result we don’t know of every single side-effect. The chance to report a new adverse reaction arises from the large number of patients across the world who are treated with those drugs. Adverse reactions are organized in terms of System Organ Class (SOC) by MedDRA and the frequency is defined as follows: (1) very common (≥1/10), common (≥1/100 to <1/10), uncommon (≥1/1000 to <1/100), rare (≥1/10,000 to <1/1000), very rare (<1/10,000), and the last, not known, which means that the frequency cannot be estimated from the available data. For example, the frequency of tinnitus as an adverse reaction following vaccination with the Janssen vaccine is between ≥1/10,000 to <1/1000 (rare). Additionally, a few publications infer a cause–effect relationship between Sudden Sensorineural Hearing Loss (SSNHL) and vaccination, but this relationship is described as indirect, as a result the relationship between the effects and caused must be proved and documented [22,23]. The frequency of dizziness as a side-effect after using tocilizumab is categorized as a common side-effect. On the other hand, Paxlovid (PF-07321332/ritonavir) as an inhibitor of cytochrome CYP3A, may cause many interactions by increasing the plasma concentration of many other medical products that are metabolized by CYP3A. The risk is elevated when Paxlovid is coadministered with drugs that are extensively metabolized by cytochrome CYP3A, especially those which have a high first-pass metabolism [64].

Ototoxicity relates not only to sensorineural hearing loss, but can also cause tinnitus and involve toxicity to the vestibule and cochlea. Adverse reactions can occur after long-term administration of a drug or occur as a secondary effect. It is known that recovery of a person’s health is paramount when there is the risk of death, but it must be remembered that after a therapy ends, any side-effects of the therapies may be irreversible, and these may burden them for the rest of their life and affect their mental health and quality of life. The audio-vestibular disorder such as dizziness, vertigo, hearing loss, or tinnitus may be linked to the COVID-19 and the main theories that are postulated are: (1) cochleitis or neuritis (viral involvement of the vestubulocochlear nerve or the inner ear), (2) production of the proinflammatory cytokines, (3) microvascular injuries in the area of the central and peripheral nervous system caused by the endothelial dysfunction, (4) vascular disorders that may affect cochlea and semicircular canals by ischemia, and (5) damage to the inner ear by accident after cross-reactions of T-cells or antibodies [95]. Consequently, the eventual connection between the possible audio-vestibular side-effect of drugs against COVID-19, should be discussed with similar effects that may be caused by the virus itself.

In 2021, the FDA and EMA approved monoclonal antibodies and oral drugs for treatment of COVID-19. The most frequent adverse reactions reported during clinical trials and later publications were, at least in the area of audiology and otorhinolaryngology, dizziness, blurry vision with dizziness, nasopharyngitis, and dysgeusia. As far as vaccines are concerned, dizziness as an ototoxic effect will only occur in hypersensitive people as part of anaphylactic shock, and as an adverse reaction with unknown frequency. Undoubtedly, new adverse effects will be discovered as large numbers of people are treated with COVID-19 medications and vaccines, so any knowledge gained from audiological tests in the course of monitoring for audio-vestibular disorders may be useful. Additionally, it will be useful for patients suffering the direct effects of COVID-19 [21].

Currently available audiological tests make it possible to quickly determine possible drug ototoxicity. The techniques and testing schedules can be adapted depending on the drug used, the age, the health of the patient, and their ability to undertake behavioral and audiological tests. Importantly, some audiological tests can be performed on unconscious patients or those in a pharmacological coma. For the most informative data, it is best if patients are tested before and after pharmacotherapy so that clear comparisons can be made. The gold standards in ototoxicity testing are the transient evoked otoacoustic emission test (TEOAE) and the distortion product otoacoustic emission test (DPOAE). Additionally, when it is possible, PTA (pure tone audiometry) is useful for diagnosis of hearing loss. These diagnostic methods provide a quick assessment of high frequency cochlear function. Clinical studies confirm that there is a strong relationship between otoacoustic emissions and the ototoxicity of a drug. TEOAE and DPOAE tests allow ototoxic effects to be detected at a very early stage—from the very beginning of treatment and sometimes even before any audiometric deficit can be recorded [96]. Over the past decades, three main approaches to ototoxicity monitoring via audiology have emerged: otoacoustic emissions (OAEs), high frequency audiometry (HFA), and basic audiological assessment. The superiority of the first and the second assessments has been confirmed in clinical trials. Audiological evaluations using various diagnostic tests can be used together or separately. When drugs with known ototoxic potential are used, basic tests should be performed before and after treatment with the drug, although unfortunately this is not often possible. Audiological evaluation is especially important for the elderly and remains an important part of ototoxicity monitoring [97,98,99]. However, basic audiological assessment is limited to less than 8 kHz and so cannot detect ototoxicity at the earliest stage in all patients. ABR (auditory brainstem response) audiometry is one of the most sensitive tests for detecting the very early stages of pharmaceutical-induced cochlear damage while the changes are still reversible. The ABR threshold test is a method that allows the hearing thresholds of children to be assessed who are too small, or in a health condition, that does not allow a conventional audiological behavioral test to be performed. An ABR test can be performed in patients who are asleep, unconscious, or in a pharmacological coma [100]. The reversibility of ototoxicity detected in the treatment of COVID-19 is unknown, and similarly with the audio-vestibular side-effects. However, it is strongly suggested that therapy be discontinued when abnormal audiological test results occur so as to reduce the further risk of ototoxicity, although, again, this is often difficult or impossible in situations of life or death. According to published clinical studies, the earliest adverse effects of ototoxic drugs tend to be seen as diminished responses of the outer cells of the basal cochlear turn—i.e., at high frequencies. High frequency audiometry (HFA) is an audiological assessment that involves an air conduction threshold test for frequencies above 8 kHz (in the range 16 to 20 kHz), which is more effective in detecting patients with hearing loss compared to conventional audiometry [101,102,103,104,105,106,107,108]. However, elderly patients with hearing loss may not have measurable hearing thresholds at such high frequencies, which means that a broader spectrum of diagnostic tests may need to be considered [109,110,111]. Ototoxicity tends to be systematic (i.e., proceeding from high to low frequencies) and is usually bilaterally symmetric. The OAEs most commonly used in clinical practice are TEOAEs and DPOAEs, and both can be used to detect ototoxic changes. According to the literature, DPOAEs can detect ototoxic changes earlier than TEOAEs, essentially because DPOAEs can be measured at higher frequencies than TEOAEs. Another advantage is that DPOAEs can often be recorded in the presence of quite severe sensorineural hearing loss [112,113,114,115,116]. Even though testing for HFA thresholds is pre-recorded than OAEs, OAE testing continues to be useful as part of ototoxicity monitoring as it does not require a behavioral response and is quick [17,27]. According to the American Academy of Audiology (AAA), the two most widely used ‘adverse event’ scales for hearing are the ototoxicity grades of the National Cancer Institute (NCI) Common Terminology Criteria for Adverse Events (CTCAE), and Brock’s Hearing Loss Grades. The CTCAE ototoxicity grades are as follows: grade 1 for children and adults: threshold shift or loss of 15–25 dB relative to baseline, averaged at two or more contiguous frequencies in at least one ear; grade 2 for children: threshold shift or loss of >25–90 dB, averaged at two contiguous test frequencies in at least one ear, a hearing loss sufficient to indicate therapeutic intervention, including hearing aids (e.g., >20 dB bilateral HL in the speech frequencies; >30 dB unilateral HL; and requiring additional speech/language related services); grade 3 for adults: >25–90 dB, averaged at three contiguous test frequencies in at least one ear); grade 3 for children: means an indication for cochlear implantation (CI) and requiring additional speech/language-related services; grade 4 for adults: profound bilateral hearing loss >90 dB HL). Patients treated with pharmaceuticals having recognized hearing damage potential, cancer patients receiving cisplatin-containing chemotherapy, or patients treated with aminoglycosides are automatically provided with standard audiological additional care. Patients with renal impairment, children under 3 years of age, people over 65 years of age, pregnant women, and patients who have been treated with ototoxic drugs or who will be administered an ototoxic drug for more than 14 days are also at risk [26,117].

As reported at the beginning of 2020, ototoxicity was a common feature of newly tested substances used against COVID-19. In the initial stage, promoted treatments included chloroquine and hydroxychloroquine, which eventually turned out to be ineffective and had ototoxic potential. However, there are now other drugs with possible ototoxic potential. By analyzing new, COVID-19 therapies, including those already approved by the regulatory authorities (EMA and FDA), we will be able to gain knowledge about new disease treatment protocols and their possible side-effects, including those related to the hearing organ. The earlier we can implement monitoring measures the better.

## 5. Conclusions

Following COVID-19 treatment, clinical trials and publications show that the most frequently reported adverse reactions (at least in terms of the audio-vestibular disorders found within the area of audiology and otorhinolaryngology) were dizziness, blurry vision with dizziness, nasopharyngitis, dysgeusia, and tinnitus. As far as vaccines are concerned, dizziness as an ototoxic effect may occur only in hypersensitive people as a result of anaphylactic shock, and is a rare adverse reaction. The ototoxicity of the drugs presented here does not have such severe symptoms as some drugs used in the treatment of COVID-19 in 2020 (in particular, hydroxychloroquine) and relates mainly to disorders of the vestibulocochlear system. However, there is still a need to monitor possible ototoxic side-effects arising from interactions with other ototoxic drugs. Additionally, the SARS-CoV-2 itself may cause similar audio-vestibular disorders. Many of the drugs approved by the EMA and FDA are new, and as a result not every side-effect is known. The methods presented here for audiological testing allow quick determination of drug ototoxicity.

## Figures and Tables

**Table 1:** Summary of potential ototoxicity and audio-vestibular disorders as an adverse reaction to the prevention and treatment of COVID-19 (based on the publications [40,46,51,59,61,76,78,79,80,81,82,83,84,85,86,87,88,89,90,91,92,93] and summaries of product characteristics—SmPC) [8,39,44,45,55,56,58,60,62,64,74,75,77].

Name of Medication: Anti-COVID-19 Treatment (Monoclonal Antibodies, Anti-Inflammatory Treatment)	Population	Route of Administration	Dose and Dosage	Ototoxicity as an Adverse Reaction and the Source of Information	Clinical Trial Information
Dexamethasone Phosphate	Adults and children from 12 years of age and weighing at least 40 kg, who received supplemental oxygen therapy	Orally Or Injection Or Infusion (drip) into a vein	6 mg once a day for up to 10 days	Blurry Vision with Dizziness (SmPC)	
				Publication: RECOVERY clinical study [78]	2104 patients in dexamethasone group and 4321 to receive usual care
Anakinra	The treatment of coronavirus disease 2019 (COVID-19) in adult patients with pneumonia requiring supplemental oxygen (low- or high-flow oxygen) who are at risk of progressing to severe respiratory failure	Subcutaneous injection	100 mg administered once a day	No Adverse Reactions Identified as Audio-Vestibular Disorders (SmPC)	
				and publications:	594 adult patients with COVID-19 at risk of progressing to respiratory failure (189 patients were allocated to the placebo arm; and 405 patients were allocated to the anakinra arm) [40].
				-publication:	116 patients: 59 were assigned to the anakinra group, and 57 were assigned to the usual care group [79].
Bamlanivimab	Adults and pediatric patients > 12 years old, weight > 40 kg, and at high risk for progressing to severe disease and/or hospitalization	Intravenously	Single 700-mg IV infusion over at least 60 min	Dizziness (frequency: common) (SmPC)	BLAZE-1 clinical trial
				Publication:	1035 patients (the dose: 2800 mg of bamlanivimab and 2800 mg of etesevimab, administered together) or placebo within 3 days after a laboratory diagnosis of SARS-CoV-2 [46].
Casirivimab and Imdevimab	Adults and pediatric patients (12 years of age or older weighing at least 40 kg)	Administered together by intravenous (IV) infusion	1200 mg of casirivimab and 1200 mg of imdevimab administered as a single intravenous infusion over at least 60 min as soon as possible after positive Vidal test for SARS-CoV-2 and within 10 days of symptom onset	Limited Data about Ototoxicity (SMPC)	Randomized, double-blind, placebo-controlled clinical trial in 799 non-hospitalized adults with mild to moderate COVID-19 symptoms.
				and publications:	5197 adult participants; adverse events occurred in 35% and 34% of participants administered casirivimab and imdevimab) and placebo, respectively, and injection site reactions occurred in 2.4% and 2.1% of participants, respectively [59].
				Publication:	1355 patients in the regen-cov group and 1341 patients in placebo group [80].
				publication:	753 participants in the regen-cov group and 752 participants in the placebo group [51].
				publication:	275 adult participants [81].
Tocilizumab	Critically ill patients, aged> 18 years	IV infusion via central or peripheral line over a 1-h period	Single dose of 8 mg/kg estimated or measured body weight, with a maximum total dose of 800 mg	NASOPHARYNGITIS, DIZZINESS (SmPC)	Randomized, Embedded, Multifactorial Adaptive Platform Trial for Community Acquired Pneumonia (REMAP-CAP).
				Publication:	no adverse reaction in the area of ENT was reported. serious adverse events occurred in 128 (29.8%) tocilizumab plus remdesivir and 72 (33.8%) placebo plus remdesivir patients; 78 (18.2%) and 42 (19.7%) patients, respectively, died by day 28 [82].
				-publication:	389 patients, (249 patients in the tocilizumab group and 128 patients in the placebo group) [83].
				-publication:	243 adult patients [84].
				publication:	2046 patients who had severe disease had undergone randomization in at least one remap-cap domain and 895 had undergone randomization in the immune modulation therapy domain [85].
Tixagevimab and Cilgavimab	The pre-exposure prophylaxis of coronavirus disease 2019 (COVID-19) in adults and pediatric individuals (12 years of age and older weighing at least 40 kg	Intramuscular injections	Single injection of 150 mg tixagevimab and 150 mg cilgavimab	No adverse reactions identified as audio-vestibular disorders (SmPC)	4220 subjects in two clinical trials, phase III, PROVENT and STORM CHASER
Sotrovimab	The treatment of adults and adolescents of age above 12 years and weighing minimum 40 kg, suffering from COVID-19 disease and not requiring supplemental oxygen and at the same time are at risk of progressing to severe phase of COVID-19	Infusion	500 mg of sotrovimab in 8 mL (62.5 mg/mL) in 50 mL or 100 mL of sodium chloride (0.9%) or 5% glucose	No adverse reactions identified as audio-vestibular disorders (SmPC)	clinical trial (COMET-ICE) of 1057 subjects
				publication:	583 patients (291 in the sotrovimab group and 292 in the placebo group [61]
Sarilumab	Critically ill patients, aged> 18 years Adults and pediatric patients >16 years of age	IV infusion through peripheral or central line over a 1-h period	Single dose of 400 mg	NASOPHARYNGITIS (SmPC)	Sarilumab COVID-19 clinical trial
**Name of Medication and Source of Information Vaccines**	**Population**	**Route of Administration**	**Dose and Dosage**	**Ototoxicity as an Adverse Reaction**	**Clinical Trial Information**
Pfizer + Biontech Vaccine: Comirnaty	Adults and children above 5 years old	One dose (0.3 mL) contains 30 µg of COVID-19 mRNA embedded in lipid nanoparticles for adults and 10 µg/dose for children	Intramuscularly—twice with an interval of 21 days between doses	Dizziness may occur only in hypersensitive people as apart of anaphylactic shock and as adverse reaction (SmPC)	Randomized, Embedded, Multifactorial Adaptive Platform Trial for Community Acquired Pneumonia (REMAP-CAP) 2 clinical studies that include 21,744 participants, who received at least one dose of vaccine
				Dizziness and nasal stuffiness	1245 participants; the aim of the study was to assess the safety and side-effects of the bnt162b2 mRNA vaccine for coronavirus disease 2019 (COVID-19) [86].
				-publication:	890,828 persons; 42-day follow-up; aim: evaluation of the safety of the bnt162b2 mRNA vaccine [87].
				Publication:	43,448 patients (above 16 years old): 21,720 with bnt162b2 and 21,728 with placebo. -adverse reactions: the safety profile of bnt162b2 was characterized by short-term, mild-to-moderate pain at the injection site, fatigue, and headache. the incidence of serious adverse events was low and was similar in the vaccine and placebo groups [88].
COVID-19 Vaccine Moderna	Adults and children above 5 years old	One dose (0.5 mL) contains 100 µg of COVID-19 messenger RNA embedded in lipid nanoparticles for adults and 50 µg/dose for children	Intramuscularly—twice with an interval of 28 days between the doses	Dizziness may occur only in hypersensitive people as apart of anaphylactic shock and as adverse reaction (SmPC)	Clinical Phase 3 trial (randomised, placebo controlled, observer blind) involving 30,351 participants who received at least one dose of vaccine
COVID-19 Vaccine AstraZeneca	Adults	One dose (0.5 mL) contains COVID-19 vaccine (ChAdOx 1-S* recombinant) 5 × 10^10^ viral particles	Intramuscularly—twice with an interval of 28 days between two doses (from 4 to 26 weeks)	Dizziness may occur only in hypersensitive people as apart of anaphylactic shock and as adverse reaction (SmPC)	4 clinical trials involving; 23,745 participants; Adverse reaction: dizziness may occur only in hypersensitive people as apart of anaphylactic shock and as adverse reaction.
				publication:	43,788 participants underwent randomization and received vaccine or placebo, and 39,185 participants who were seronegative for SARS-CoV-2 at baseline were included in the per-protocol analysis population for the double-blind phase [89].
				publication:	adverse reactions: unsolicited adverse events were recorded for all participants for 28 days after each dose of azd1222 or placebo, but no in the area of ENT [76]
				publication:	175 severe adverse events occurred in 168 participants, 84 events in the chadox1 ncov-19 group, and 91 in the control group, but none in the area of ENT [90].
				Publication:	the incidence of serious adverse events was balanced between the two groups. Three deaths occurred in the vaccine group (none were COVID-19-related), and 16 in the placebo group (5 were COVID-19-related) [91].
Covid-19 Vaccine Novovax (Nuvaxovid)	Adults	One dose (0.5 mL) contains 5 µg of SARS-CoV-2 spike protein and is adjuvanted with Matrix-M	Intramuscularly—twice with an interval of 28 days between the doses	dizziness may occur only in hypersensitive people as apart of anaphylactic shock and as adverse reaction (smpc)	5 clinical trials. Total number of patients enrolled to this study was 49,950 adult participants (Nuvaxovid group *N* = 30,058 and placebo group *N* = 19,892)
Covid-19 Vaccine Janssen	Adults	Janssen is an adenovirus vaccine against COVID-19 diseases and one dose (0.5 mL) contains adenovirus type 26 encoding the SARS-CoV-2 spike glycoprotein (Ad26.COV2-S), not less than 8.92 log10 infectious units	Intramuscularly—at least 2 months after the primary vaccination	dizziness, tinnitus (smpc)	The safety of COVID-19 Vaccine Janssen was evaluated in an ongoing Phase 3 study (COV3001), with a total of 21,895 adults
Name of Medication and Source of Information Oral Treatment	Population	Route of Administration	Dose and Dosage	Ototoxicity as an Adverse Reaction	Clinical Trial Information
PF-07321332 and Ritonavir (Brand Name: Paxlovid)	Adults	300 mg (two tablets of 150 mg each) of nirmatrelvir and 100 mg (one tablet) of ritonavir	Orally, twice a day for 5 days	dysgeusia, as a side effect in the area of ent (This side-effect was reported by 6% of subjects from Paxlovid group in comparison with the 1% in the placebo group) (SmPC)	The safety and adverse reactions were assessed in a clinical trial (phase 2/3, randomized, placebo-controlled (C4671005 EPIC-HR)), 2224 adults
				Publication:	-adverse events: that emerged during the treatment period was similar in the two groups (any adverse event, 22.6% with nirmatrelvir plus ritonavir vs. 23.9% with placebo; serious adverse events, 1.6% vs. 6.6%; and adverse events leading to discontinuation of the drugs or placebo, 2.1% vs. 4.2%), dysgeusia (5.6% vs. 0.3%) [92]
Molnupiravir	adults	800 mg	orally, twice a day for 5 days	dizziness (smpc)	Phase 3, double-blind, acronym MOVe-OUT), 1411 adults
				publication:	adverse reactions were reported in 30.4% participants in the molnupiravir group and 33% of participants in the placebo group [93]

## Data Availability

The data presented in this study are available on request from the corresponding author.
